# Risk factors, prenatal diagnosis, and outcome of posterior placenta accreta spectrum disorders in patients with placenta previa or low‐lying placenta: A multicenter study

**DOI:** 10.1111/aogs.15132

**Published:** 2025-05-19

**Authors:** The ADoPAD (Antenatal Diagnosis of Placental Attachment Disorders) study group, Francesco D’Antonio, Alessandro Lucidi, Nicola Fratelli, Claudia Maggi, Cecilia Cavalli, Andrea Sciarrone, Danilo Buca, Anna Garofalo, Elsa Viora, Patrizia Vergani, Sara Ornaghi, Marta Betti, Isadora Vaglio Tessitore, Anna Franca Cavaliere, Silvia Buongiorno, Annalisa Vidiri, Elisa Fabbri, Enrico Ferrazzi, Valeria Maggi, Irene Cetin, Tiziana Frusca, Tullio Ghi, Christine Kaihura, Elvira Di Pasquo, Tamara Stampalija, Chiara Belcaro, Mariachiara Quadrifoglio, Michela Veneziano, Federico Mecacci, Serena Simeone, Anna Locatelli, Sara Consonni, Nicola Chianchiano, Francesco Labate, G. Calcagno, Antonella Cromi, Fabio Ghezzi, Emma Bertucci, Fabio Facchinetti, Giulia Andrea Giuliani, Anna Fichera, Daniela Granata, Francesca Foti, Laura Avagliano, Gaetano Pietro Bulfamante, Asma Khalil, Maria Elena Flacco, Lamberto Manzoli, Federico Prefumo, Giuseppe Calì

**Affiliations:** ^1^ Center for Fetal Care and High‐Risk Pregnancy, Department of Obstetrics and Gynecology University of Chieti Chieti Italy

**Keywords:** outcome, placenta accreta spectrum, ultrasound

## Abstract

**Introduction:**

Placenta accreta spectrum (PAS) disorders occur when the definitive placenta develops within the uterus scar area. Although classically PAS develops in the anterior wall of the uterus mainly, it can also develop in the posterior uterine wall. The aim of this study was to report the risk factors, diagnostic accuracy of prenatal imaging, and surgical outcome of pregnancies complicated by posterior PAS in women with placenta previa or low‐lying.

**Material and Methods:**

Secondary analysis of a multicenter prospective study involving 16 referral hospitals in Italy (ADoPAD Study). Inclusion criteria were patients with a posterior low‐lying placenta (<20 mm from the internal cervical os) or placenta previa (covering the os), aged ≥18 years undergoing ultrasound assessment at ≥26^+0^ weeks of gestation. The reference standard for PAS was represented by the failure of placental separation at delivery or by pathological analysis. The primary aim was to report the risk factors associated with the occurrence of posterior PAS. The secondary aims were to evaluate the ability of prenatal ultrasound in detecting posterior PAS and to report its surgical outcome compared to posterior placental previa or low‐lying with no PAS and anterior PAS, respectively, and in patients with a prenatal compared to post‐natal diagnosis. Univariate and diagnostic accuracy analyses were used to analyze the data.

**Results:**

258 patients were included in the analysis. Posterior PAS occurred in 8.1% (*n* = 21; 95% CI 5.4–12.1) of patients. There was a higher incidence of one or more prior CS (62% vs. 21%, *p* < 0.001) and myomectomy with uterine penetration (71.0% vs. 3.4%, *p* < 0.001) in patients with posterior PAS compared to those with no PAS. In patients with posterior PAS, placenta accreta occurred in 66.67% (14/21), increta in 23.81% (5/21), and percreta in 9.52% (2/21) of cases. Posterior PAS confirmed at birth was diagnosed prenatally by ultrasound in 62% (13/21) of cases. When comparing anterior with posterior PAS, patients with anterior PAS were more likely to have a prior CS (82% vs. 62%; *p* = 0.0049) and placenta percreta (54% vs. 10%; *p* < 0.001). Finally, the need for hysterectomy (89% vs. 48%; *p* < 0.001) was higher, while that of balloon tamponade insertion was lower (17% vs. 52%; *p* = 0.001) in patients with anterior compared to posterior PAS.

**Conclusions:**

Prior uterine surgery in patients with placenta previa or low‐lying represents the commonest risk factors for posterior PAS. The diagnostic accuracy of ultrasound in detecting posterior PAS is lower in cases with posterior compared to anterior PAS. Finally, in referral centers, posterior PAS disorders were associated with a lower risk of hysterectomy compared to anterior PAS.

## Graphical abstract

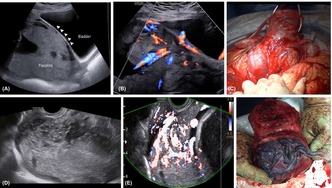

**Abbreviations**
CI: confidence interval
CS: cesarean section
MRI: magnetic resonance imaging
PAS: placenta accreta spectrum

**Key message**

Prenatal ultrasound has a low diagnostic accuracy in detecting posterior compared to anterior placenta accreta spectrum disorder. However, posterior placenta accreta spectrum disorders are associated with a lower risk of hysterectomy compared to cases with anterior implantation.

## 1 INTRODUCTION

Placenta accreta spectrum (PAS) disorders encompass a heterogenous group of conditions which occur when the gestational sac implants and the definitive placenta develops within the uterine scar area.^1^, ^2^, ^3^

Prenatal identification of PAS disorders is crucial and is associated with a reduced risk of maternal morbidity and even mortality.^4^, ^5^, ^6^ Classically, PAS develops in the anterior uterine wall mainly as the consequence of myometrial remodeling in the area of a prior cesarean section (CS) scar.^7^ However, PAS can also develop in other areas, including the posterior uterine wall, even in patients without prior uterine scars.

A recent systematic review including 114 patients with posterior PAS reported that placenta previa, a prior CS, and curettage represent the main risk factors for posterior PAS. Prenatal identification of these disorders was lower compared to anterior PAS, and most cases presented with placenta accreta at histopathological analysis.^7^, ^8^

The aims of this study were to report the risk factors, diagnostic, and surgical outcome of pregnancies complicated by posterior PAS from a large cohort of patients with placenta previa and low‐lying placenta.

## 2 MATERIAL AND METHODS

This is a secondary analysis of a multicenter prospective observational study (Antenatal Diagnosis of Placental Attachment Disorders, ADoPAD) involving 16 Italian hospitals, conducted from October 2014 to January 2019.^9^ STROBE guidelines for cohort studies were followed.^10^ The study was registered with ClinicalTrials.gov (NCT02442518).

**FIGURE 1:**
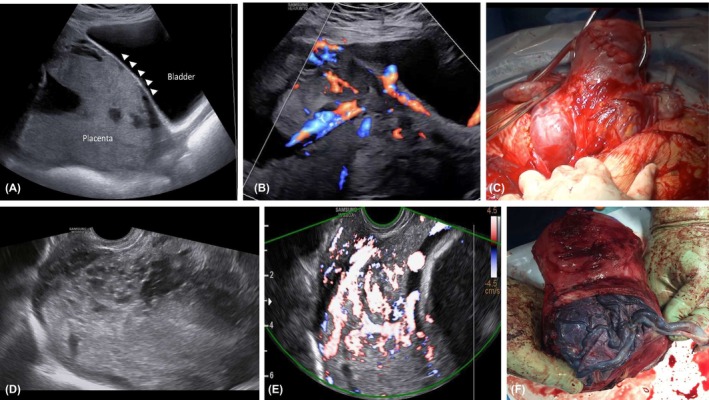
(A, B) Ultrasound signs of anterior PAS, including loss of the clear zone and the presence of a vessel running perpendicular to the uterine bladder interface and interrupting the continuity of the myometrium. The presence of a partially full bladder allows an accurate assessment of the relationship between the placenta and the myometrium. (C) Anatomical specimen of anterior PAS after hysterectomy. (D–F) Posterior PAS. (D, E) The lack of the interface provided by a full bladder does not allow accurate identification of the myometrium beneath the placenta which cannot be clearly identified through all its course. Please note the presence of lacunae. In this case, the suspicion is raised by the presence of hypervascularization of the cervical tissue. (F) Anatomical specimen after hysterectomy of a case with posterior PAS.

Inclusion criteria were patients with a posterior low‐lying placenta (<20 mm from the internal cervical os) or placenta previa (covering the os), aged ≥18 years, who underwent transabdominal and transvaginal ultrasound assessment at ≥26^+0^ weeks to assess placental location and the risk of PAS. All patients underwent a further scan at 34–36 weeks' gestation for repeat assessment of placental location. All cases were assessed using transabdominal and transvaginal ultrasound with a partially full bladder to allow assessment of the interface between the uterine serosa and the bladder wall by operators with clinical expertise in prenatal diagnosis of PAS. Ultrasound suspicion of posterior PAS was raised in the presence of at least one of these signs: obliteration of the hypoechogenic space between the uterus and the placenta and abnormal placental lacunae, defined as the presence of numerous lacunae including some that are large and irregular (Finberg Grade 3), often containing turbulent flow visible on grayscale imaging (Figure 1).^11^, ^12^, ^13^, ^14^ Furthermore, the thickness of the lower placental edge was measured within 1 cm of the meeting point of the basal and chorionic plates.

The primary aim of the present study was to report the risk factors associated with the occurrence of posterior PAS in patients with low‐lying placenta or placenta previa.

The secondary aims were to evaluate the ability of prenatal ultrasound in detecting posterior PAS and to report its surgical outcome compared to patients presenting with posterior placenta previa or low‐lying with no PAS. Furthermore, we performed sub‐group analyses comparing patients with a prenatal compared to a post‐natal diagnosis of posterior PAS and in whose with posterior compared to anterior PAS.

The reference standard for PAS was represented by the failure of placental separation at delivery or by pathological analysis. Pathological examination was performed according to a predefined protocol, with the pathologists blinded to the ultrasound findings. Pathological diagnosis of placenta accreta, whether performed on hysterectomy specimens or, in case the uterus was retained, relied on findings of placental villi in direct apposition to the myometrium in the absence of intermediate decidual layers between anchoring villi and muscular cells.^1^

According to the most recent studies, the incidence of posterior PAS disorders among a high‐risk population of women with low‐lying placenta or placenta previa was 8.1%.^9^ However, in the meta‐analysis by Tinari et al., on posterior PAS, the authors reported the figures for diagnostic accuracy only in terms of detection rate. Therefore, we based the power calculation on the diagnostic accuracy of ultrasound in detecting all types of PAS reported in the original Adopad cohort. Based upon these data, and assuming: (1) a sensitivity and a specificity of US to detect PAS of 80% and 95%,^9^ respectively; (2) an alpha‐error of 0.05, a minimum of 225 women would be required to achieve a 80% statistical power to detect an increase in the values of sensitivity and specificity ≥10%.

We first evaluated the prevalence of posterior PAS by each potential predictor using standard univariate analyses: chi‐squared test for categorical variables; *t*‐test and Kruskal–Wallis test for normally distributed and non‐normally distributed continuous variables, respectively (distribution assessed with Shapiro‐Wilk test). The overall number of detected ultrasound sign was used to generate four dichotomic variables.^15^, ^16^

Additionally, we estimated the potential of each ultrasound sign and of selected gestational characteristics (BMI >30, multigravidity, multiparity, Caucasian ethnicity, prior CS, prior curettage, prior myomectomy with uterine penetration, history of placenta previa major) in predicting posterior PAS. We thus computed summary estimates of sensitivity, specificity, positive and negative predictive values, positive and negative likelihood ratios, and diagnostic odds ratios. Similarly, we assessed the diagnostic accuracy of the presence of 1, 2, or 3 ultrasound signs.

In addition, the same approach was adopted to compare the maternal/gestational characteristics, ultrasound signs and selected peri/postpartum outcomes between patients with posterior versus anterior PAS disorders, and in those with compared without a prenatal diagnosis of posterior PAS, who were compared using standard univariate analyses. However, due to the small number of patients with posterior PAS, no meaningful multivariate analysis could be performed.

Statistical significance was defined as a two‐sided *p*‐value<0.05, and all analyses were performed using Stata 13.1 (Stata Corp., College Station, Texas, 2013).

## 3 RESULTS

**FIGURE 2:**
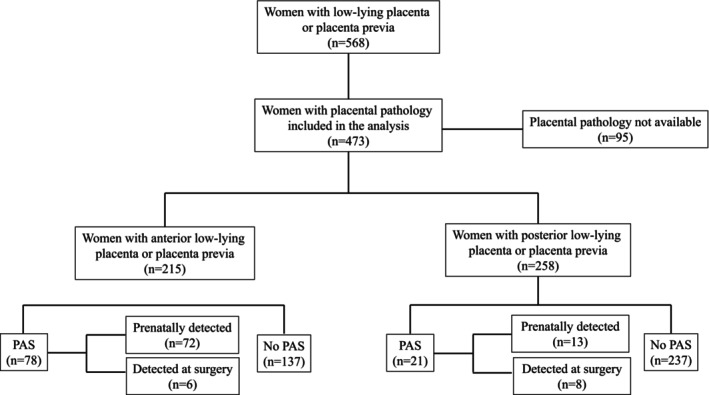
STROBE flowchart.

The original study population included 568 patients with placenta previa or low‐lying; 95 were excluded due to a lack of pathological report of the placenta, leaving 473 women available for the analysis. Out of these, 258 patients presented with posterior low‐lying or placenta previa and represented the study population included in the analysis. Posterior PAS occurred in 8.1% of patients (95% confidence interval [CI] 5.4–12.1; 21/258) in this cohort (Figure 2). Placental location was confirmed in all affected and unaffected cases at surgical exploration after hysterotomy or pathological examination in the case of hysterectomy.

Main maternal, pregnancy, and ultrasound characteristics of pregnancies with and without posterior PAS are reported in Table 1.

**TABLE 1:** Selected demographic and clinical characteristics of the sample, overall and among the patients with versus without posterior placenta accreta spectrum (PAS) disorders.

Variables	Overall sample (*N* = 258)	Posterior PAS (*N* = 21)	No PAS (*N* = 237)	*p* ^a^
Demographic and clinical characteristics				
Mean maternal age at delivery in years (SD)	35.7 (5.8)	34.7 (6.1)	35.8 (5.8)	0.4
Mean BMI, (SD)	23.5 (4.1)	24.4 (6.6)	23.5 (3.9)	0.3
Multigravid patients, %	69.0 (*N* = 178)	76 (*N* = 16)	68.4 (*N* = 162)	0.5
Nulliparous patients	48.8 (*N* = 126)	33 (*N* = 7)	50.2 (*n* = 119)	
ART, %	12.0 (*N* = 31)	10 (*N* = 2)	12.2 (*N* = 29)	0.7
Caucasian ethnicity, %	82.2 (*N* = 212)	81 (*N* = 17)	82.3 (*N* = 195)	0.8
Prior CS, %	24.4 (*N* = 63)	62 (*N* = 13)	21.0 (*N* = 50)	<0.001
Prior CS during labor, %	7.4 (*N* = 19)	14 (*N* = 3)	6.8 (*N* = 16)	0.2
Prior curettage, %	34.5 (*N* = 89)	29 (*N* = 6)	35.0 (*N* = 83)	0.6
Prior myomectomy with uterine penetration, %	8.9 (*N* = 23)	71 (*N* = 15)	3.4 (*N* = 8)	<0.001
Major placenta previa, %	73.3 (*N* = 159)	86 (*N* = 18)	72.2 (*N* = 141)	0.2
Posterior PAS disorders, % (*n*)				
Accreta	‐	66.7 (*N* = 14)	‐	‐
Increta	‐	23.8 (*N* = 5)	‐	‐
Percreta	‐	9.5 (*N* = 2)	‐	‐
US signs				
Interrupted hypoechoic retroplacental space, %	11.2 (*N* = 30)	62 (*N* = 13)	7.2 (*N* = 17)	<0.001
Interrupted hyperechogenic bladder line, %	1.8 (*N* = 5)	14 (*N* = 3)	0.8 (*N* = 2)	<0.001
Lacunae, %	15.1 (*N* = 39)	62 (*N* = 13)	11.0 (*N* = 26)	<0.001
One sign only (vs. none), %	8.9 (*N* = 23)	0 (*N* = 0)	9.7 (*N* = 23)	0.4
Two signs (vs. ≤1), %	7.4 (*N* = 19)	57 (*N* = 12)	2.9 (*N* = 7)	<0.001
Three signs (vs. ≤2), %	1.9 (*N* = 5)	14 (*N* = 3)	0.84 (*N* = 2)	<0.001

Abbreviations: ART, assisted reproductive technique; BMI, body mass index; CS, cesarean section; MRI, magnetic resonance imaging; SD, standard deviation; US, ultrasound.

^a^ Chi‐squared test for categorical variables; *t*‐test and Kruskal–Wallis test for normally distributed and non‐normally distributed continuous variables, respectively.

There was no difference in either mean maternal age (*p* = 0.4), BMI (*p* = 0.3), ethnicity (*p* = 0.8), multigravidity (*p* = 0.5), prior curettage (*p* = 0.6), or placenta previa entirely covering the internal cervical os (*p* = 0.10) in pregnancies complicated compared to those not complicated by posterior PAS, while there was a higher incidence of at least one prior CS (62% vs. 21%, *p* < 0.001) and myomectomy (71.0% vs. 3.4%, *p* < 0.001) in patients with posterior PAS compared to those with placenta previa or low‐lying and no PAS. Diagnostic accuracy of the different maternal and pregnancy characteristics in predicting posterior PAS is reported in Table S1.

In patients with posterior PAS, placenta accreta occurred in 66.67% (14/21), increta in 23.81% (5/21), and percreta in 9.52% (2/21) of cases.

Regarding the ultrasound characteristics, either the presence of interrupted retroplacental space (62% vs. 7%; *p* < 0.001), interrupted hyperechogenic bladder line (14.0% vs. 0.8%, *p* < 0.001), and placental lacunae (62% vs. 11%) were more common in patients with posterior PAS compared to those with placenta previa or low‐lying and no PAS (Table 1). The diagnostic accuracy of the different ultrasound signs, alone or in combination, in detecting posterior PAS is reported in Table 2. Overall, ultrasound showed a poor diagnostic accuracy in detecting posterior PAS. There was no difference in the diagnostic accuracy of ultrasound in detecting posterior PAS when using two or three ultrasound signs (Table 2). Overall, 62% of patients with posterior PAS confirmed at birth had a prenatal diagnosis of these anomalies by ultrasound. Only seven cases were referred to magnetic resonance imaging (MRI) and all had a suspicion of posterior PAS. However, in view of the small number of cases undergoing MRI, an objective statistical comparison between the two techniques could not be performed.

**TABLE 2:** Diagnostic accuracy of each ultrasound sign to predict a diagnosis of posterior placenta accreta spectrum (PAS) disorder: Summary estimates of sensitivity, specificity, positive and negative predictive values (PPV and NPV), positive and negative likelihood ratios (LR+ and LR−) and diagnostic odds ratios (DOR).

Clinical characteristics	Sensitivity % (95% CI)	Specificity % (95% CI)	PPV % (95% CI)	NPV % (95% CI)	LR+ (95% CI)	LR− (95% CI)	DOR (95% CI)
Interrupted hypoechoic retroplacental space	44.8 (26.4–64.3)	96.5 (93.2–98.5)	61.9 (38.4–81.9)	93.2 (89.3–96.1)	12.8 (5.82–28.3)	0.57 (0.41–0.79)	22.4 (8.28–60.9)
Lacunae	33.3 (19.1–50.2)	96.3 (92.9–98.4)	61.9 (38.4–81.9)	89.0 (84.3–92.7)	9.13 (4.05–20.6)	0.69 (0.55–0.87)	13.2 (5.10–34.1)
Interrupted hyperecogenic bladder line	14.3 (3.1–36.4)	99.2 (97.0–99.9)	60.0 (14.7–94.7)	92.9 (89.0–95.7)	16.9 (3.5–80.1)	0.86 (0.66–0.96)	19.0 (2.0–241.3)
One sign^a^	0.0 (0.0–15.4)	96.2 (92.7–98.4)	0.0 (0.0–36.9)	90.3 (85.7–93.8)	‐	1.04 (1.01–1.07)	0.0 (0.0–4.65)
Two or three signs^a^	55.6 (30.8–78.5)	96.6 (93.4–98.5)	55.6 (30.8–78.5)	96.6 (93.4–98.5)	16.3 (7.36–36.2)	0.46 (0.27–0.77)	35.5 (11.3–112)

Abbreviation: CI, confidence interval.

^a^ Presence of one, two or all three of the recorded signs.

Table 3 reports the outcome of pregnancies complicated by posterior PAS compared to those presenting with posterior placenta previa and no PAS. There was no difference in the mean gestational age at birth (*p* = 0.2), occurrence of antepartum hemorrhage (*p* = 0.9) and emergency CS between patients with compared to those without posterior PAS. Conversely, the median blood loss (1700 mL, interquartile range 900–2700 vs. 700 mL, interquartile range 500–1100; *p* < 0.001), maternal complications (5% vs. 0.8%; *p* = 0.003), placement of balloon tamponade (52% vs. 24%; *p* = 0.005), need for B‐Lynch suture (14% vs. 1%; *p* < 0.001), hysterectomy (48% vs. <1%; *p* < 0.001), uterine artery ligation (5% vs. 0%; *p* = 0.001), need for blood transfusion (57% vs. 12%; *p* < 0.001), and mean units of blood transfused (3.33 ± 4.80 vs. 0.33 ± 1.14; *p* < 0.001) were higher in patients with posterior PAS compared to those with posterior placenta previa or low‐lying and no PAS (Table 3).

**TABLE 3:** Selected outcomes, overall and among the patients with versus without posterior placenta accreta spectrum (PAS) disorders.

Variables	Overall sample (*N* = 258)	Posterior PAS (*N* = 21)	No PAS (*N* = 237)	*p* ^a^
Mean gestational age at delivery in weeks (SD)	35.7 (2.7)	34.8 (2.1)	35.8 (2.7)	0.2
Antepartum hemorrhage, %	43.4 (*N* = 111)	43 (*N* = 9)	43.4 (*N* = 102)	0.9
Cesarean section, %	96.1 (*N* = 248)	100 (*N* = 21)	95.8 (*N* = 227)	0.3
Emergency CS,^b^ %	34.5 (*N* = 89)	38 (*N* = 8)	34.4 (*N* = 81)	0.7
Mean birthweight in grams (SD)	2627 (636)	2235 (567)	2661 (631)	0.003
Median PPH in mL (IQR)	800 (500–1200)	1700 (900–2700)	700 (500–1100)	<0.001
Need for blood transfusion, %	15.9 (*N* = 419	57 (*N* = 12)	12.2 (*N* = 29)	<0.001
Mean Units of FFP transfused (SD)	0.58 (1.91)	3.33 (4.80)	0.33 (1.14)	<0.001
Bladder lesions	0 (*N* = 0)	0 (*N* = 0)	0 (*N* = 0)	1.000
Postpartum Bakri balloon placement, %	26.4 (*N* = 66)	52 (*N* = 11)	24.1 (*N* = 57)	0.005
Postpartum B‐Lynch suture, %	1.9 (*N* = 5)	14 (*N* = 3)	0.8 (*N* = 2)	<0.001
Hysterectomy, %	4.3 (*n* = 11)	48 (*N* = 10)	0.4 (*N* = 1)	<0.001
Uterine artery ligation, %	0.4 (*N* = 1)	5 (*n* = 1)	0.0 (*N* = 0)	0.001
Interventional radiology, %	1.2 (*N* = 3)	5 (*N* = 1)	0.8 (*N* = 2)	0.11
Maternal death, %	0.0 (*N* = 0)	0 (*N* = 0)	0 (*N* = 0)	‐

Abbreviations: CS, cesarean section; FFP, fresh frozen plasma; IQR, interquartile range; PPH, postpartum hemorrhage; SD, standard deviation.

^a^ Chi‐squared test for categorical variables; *t*‐test and Kruskal–Wallis test for normally distributed and non‐normally distributed continuous variables, respectively.

^b^ Due to: bleeding, fetal growth restriction, HELLP syndrome, intra‐uterine death, placental abruption, uterine contraction.

In the present cohort, PAS occurred in 36.3 (95% CI 30.2–42.9) in with patients with anterior and in 8.1% (95% CI 5.4–12.1) in those with posterior PAS (Table 4).

**TABLE 4:** Selected outcomes, overall and among the patients with posterior versus anterior placenta accreta spectrum (PAS) disorders.

	Overall sample (*N* = 99)	Posterior PAS (*N* = 21)	Anterior PAS (*N* = 78)	
Variables	(*N* = 86)	(*N* = 18)	(*N* = 68)	*p* ^a^
Mean gestational age at delivery in weeks (SD)	34.3 (2.3)	34.8 (2.1)	34.2 (2.3)	0.3
Antepartum hemorrhage, %	35.4 (*N* = 35)	43 (*N* = 9)	30.7 (*N* = 24)	0.3
Cesarean section, %	99.0 (*N* = 98)	100 (*N* = 21)	98.7 (*N* = 77)	0.6
Emergency CS,^b^ %	26.3 (*N* = 26)	38 (*N* = 8)	22.5 (*N* = 18)	0.05
Mean birthweight in grams (SD)	2314 (485)	2235 (567)	2336 (462)	0.4
Median PPH in mL (IQR)	1500 (700–2700)	1700 (900–2700)	1500 (650–2500)	0.5
Need for blood transfusion, %	55.6 (*N* = 55)	57 (*N* = 12)	55.2 (*N* = 43)	0.9
Mean Units of FFP transfused (SD)	3.5 (5.3)	3.3 (4.8)	3.5 (5.4)	0.9
Bladder lesions	4.0 (*N* = 4)	0 (*N* = 0)	5.2 (*N* = 4)	0.573
Postpartum Bakri balloon placement, %	24.2 (*N* = 24)	52 (*N* = 11)	16.7 (*N* = 13)	0.001
Postpartum B‐Lynch suture, %	8.1 (*N* = 8)	14 (*N* = 3)	6.4 (*N* = 5)	0.2
Hysterectomy, %	79.8 (*N* = 79)	48 (*N* = 10)	88.5 (*N* = 69)	<0.001
Uterine artery ligation, %	11.1 (*N* = 11)	5 (*n* = 1)	12.8 (*N* = 10)	0.3
Interventional radiology, %	11.1 (*N* = 11)	5 (*N* = 1)	12.8 (*N* = 10)	0.3
Maternal death, %	0 (*N* = 0)	0 (*N* = 0)	0.0 (*N* = 0)	‐

Abbreviations: CS, cesarean section; FFP, fresh frozen plasma; IQR, interquartile range; PPH, postpartum hemorrhage; SD, standard deviation.

^a^ Chi‐squared test for categorical variables; *t*‐test and Kruskal–Wallis test for normally distributed and non‐normally distributed continuous variables, respectively.

^b^ Due to: bleeding, FGR, HELLP syndrome, IUD, placental abruption, uterine contraction.

There was no difference in mean maternal BMI (*p* = 0.6), parity (*p* = 0.14), ethnicity (*p* = 0.4), rate of pregnancies conceived by assisted reproductive techniques (*p* = 0.5), while patients with anterior PAS were more likely to have a prior CS (82% vs. 62%; *p* = 0.0049) (Table S2). Patients with anterior PAS had also a higher risk of presenting with placenta percreta (54% vs. 10%; *p* < 0.001). Regarding the distribution of ultrasound signs, patients with anterior PAS were more likely to present interruption of the hypoechoic retroplacental space (90% vs. 62%; *p* = 0.002) and interruption of the hyperechogenic bladder line (68% vs. 14%; *p* < 0.001) compared to posterior PAS, while there was no difference in the presence of intra‐placental lacunae (*p* = 0.5) between the two types of PAS disorders. Prenatal diagnosis by ultrasound detected all cases affected by anterior compared to 62% of those with posterior PAS. Finally, the need for hysterectomy (89% vs. 48%; *p* < 0.001) was higher, while that of emergency CS (38% vs. 23%; *p* = 0.005) and balloon tamponade insertion was lower (52% vs. 17%; *p* = 0.001) in patients with anterior PAS, while there was no difference in the mean maternal blood loss (*p* = 0.5), bladder lesions (*p* = 573), need for transfusion (*p* = 0.9), units of blood transfused (*p* = 0.9), and need for uterine artery ligation (*p* = 0.3) between patients with anterior compared to posterior PAS.

Finally, we compared maternal outcome in patients with posterior PAS diagnosed prenatally versus at the time of surgery (Table 5). The analysis was affected by a very small number of included cases, showing no difference in the risk of emergency CS, postpartum hemorrhage, need for blood transfusions, or mean units of blood products transfused between the two groups, while the risk of hysterectomy was higher in patients with a prenatal diagnosis of posterior PAS (Table 5).

**TABLE 5:** Selected outcomes among women with prenatally detected versus prenatally undetected placenta accreta spectrum (PAS) disorders.

Variables	Prenatally detected PAS (*N* = 13)	Prenatally undetected PAS (*N* = 8)	*p* ^a^
Antepartum hemorrhage, % (*n*)	46.2 (6)	37.5 (3)	0.7
Emergency CS,^b^ % (*n*)	76.9 (10)	100 (8)	0.14
Median PPH in mL (IQR)	1500 (700–3000)	1750 (1000–2450)	0.6
Need for blood transfusion, % (*n*)	61.5 (8)	50.0 (4)	0.6
Median Units of FFP transfused (IQR)	2.0 (0–5)	1.0 (0–3)	0.6
Hysterectomy, % (*n*)	69.2 (9)	12.5 (1)	0.011

Abbreviations: CS, cesarean section; FFP, fresh frozen plasma; IQR, interquartile range; PPH, postpartum hemorrhage.

^a^ Chi‐squared test and Kruskal–Wallis test for categorical and continuous variables, respectively.

^b^ Due tobleeding, fetal growth restriction, HELLP syndrome, intra‐uterine death, placental abruption, uterine contraction.

## 4 DISCUSSION

The findings from this study show that prior CS and myomectomy were the commonest risk factors associated with the occurrence of posterior PAS in women with placenta previa or low‐lying placenta. Ultrasound detected only 60% of cases affected by posterior PAS, while it was not possible to elucidate the role of MRI in view of the small number of cases included in this cohort who had placental MRI. Most pregnancies with posterior PAS disorders present with placenta accreta, while the occurrence of placenta percreta was significantly lower compared to anterior PAS. Finally, in referral centers, posterior PAS was associated with a lower risk of hysterectomy compared to anterior PAS.

Only a few studies reported the risk factors, outcome, and prenatal diagnosis of posterior PAS. A recent meta‐analysis by Tinari et al. including 20 studies and 114 cases of posterior PAS reported that placenta previa, prior uterine surgery, and multiparity were the strongest risk factors for PAS. The large majority of cases with posterior PAS in the present cohort were placenta accreta at histopathological analysis, while only 10% of cases were percreta. These findings are in line with those reported by this meta‐analysis by Tinari et al. and confirm that the risk of PAS increases in patients with prior uterine scarring and the lower diagnostic accuracy of ultrasound compared to anterior PAS.^7^

Risk stratification for PAS disorders is crucial to maximize the diagnostic accuracy of prenatal imaging in detecting these anomalies. Placenta previa and prior CS or uterine surgery represent the classical risk factors for PAS when the placenta develops within the prior cesarean section scar.^5^ Prenatal identification of PAS is crucial to reduce the burden of maternal morbidity and even mortality associated with these anomalies as it allows referral to centers with a high expertise in their management. Prenatal ultrasound has a high diagnostic accuracy in detecting anterior PAS, while MRI is commonly used to confirm the diagnosis and allows an accurate description of the topography of placenta implantation, which in turn may help in adopting a tailored surgical management.^17^, ^18^, ^19^, ^20^, ^21^

In the present study, the strongest risk factors associated with posterior PAS in patients with placenta previa were prior CS and myomectomy.

The presence of myomectomy with uterine penetration in the posterior wall of the uterus may potentially lead to similar changes observed in patients with anterior PAS developing in the CS scar area. In such cases, the loss and remodeling of the normal uterine wall structure following CS allows the extra‐villous trophoblast to reach and contribute to the transformation of large peripheral uterine arteries under the scar area.^20^ Continuous high‐pressure arterial intervillous flow is probably the main factor for the increase in fibrinoid deposition at the utero‐placental interface with progressive distortion of the overlying cotyledons and the development of PAS. However, most posterior PAS present with milder types of abnormal placental implantation, with placenta percreta occurring in about 10% only compared to 54% of cases of anterior PAS in the ADoPAD cohort. The reason for the reduced risk of placenta percreta in patients with posterior compared to anterior PAS but may rely on the fact that the incision on the anterior wall of the uterus during the CS is always performed in the lower uterine segment which presents a reduced thickness compared to the non‐pregnant uterus undergoing myomectomy and this may affect the risk of niche formation. Furthermore, the posterior wall of the uterus is usually thicker than the anterior wall, which undergoes a significant thinning during labor, thus potentially affecting its integrity after surgical incision. Finally, the closure of a myomectomy scar is commonly performed by using a combination of multiple layers of interrupted and continuous suture compared to single of double suture adopted during the CS. Establishing an association between a prior CS and posterior PAS is also challenging. PAS occurs due to uterine remodeling when the integrity of the uterine wall is altered. Anatomically, CS is not associated with damage to the posterior uterine wall, thus making this reported association difficult to explain. Unfortunately, a multivariate analysis elucidating the strength of association between CS and posterior PAS could not be performed in view of the small number of cases affected.

In the present study, ultrasound could detect posterior PAS in only 62% of cases: this lower sensitivity compared to cases of anterior PAS is in line with that reported by the meta‐analysis by Tinari et al.^7^ These findings question whether patients with posterior low‐lying or previa should be referred to detailed ultrasound assessment, although the diagnostic performance of ultrasound in detecting posterior PAS was low. In anterior PAS, the presence of a partially full bladder allows a more precise assessment of the myometrium and allows recognition of the commonest risk factors associated with PAS, including the loss of the “clear zone,” myometrial thinning, bladder wall interruption, the presence of a placental bulge, exophytic mass, or uterovesical hypervascularity.^22^, ^23^ These signs are subjectively more difficult to identify in posterior PAS in view of the lack of the anechoic area provided by the bladder. This may also explain the higher diagnostic accuracy of MRI in detecting cases with posterior PAS compared to ultrasound reported in the published literature. In this scenario, MRI assessment should be considered in patients with posterior placenta previa presenting with risk factors for PAS such as myomectomy or multiple curettage.^7^

PAS disorders are associated with a high risk of maternal surgical morbidity including hemorrhage and urological complications, thus supporting their management in centers with high expertise in their surgical management in order to reduce adverse maternal outcomes.^24^, ^25^ In the present study, the need for hysterectomy was lower in patients with posterior compared to anterior PAS, while that of emergency CS and balloon tamponade insertion higher. The lower risk of hysterectomy in patients with posterior PAS may be explained by the lower rate of placenta percreta in these patients. Despite that, about 50% of patients with posterior PAS underwent hysterectomy. The lack of difference in hemorrhagic morbidity in patients with posterior compared to anterior PAS may be explained by the fact that all patients in the ADoPAD cohort underwent surgery in referral centers with multi‐disciplinary teams with surgical expertise, which should always be considered in patients presenting with risk factors for PAS. Furthermore, the degree of uterine remodeling is commonly milder in posterior PAS as testified by the small number of cases affected by placenta percreta reported in these patients. This may induce the surgeon in adopting a more conservative approach as also testified by the higher number of balloon tamponade insertions and B‐Lynch sutures in patients with posterior compared to anterior PAS. The higher occurrence of the use of balloon tamponade and B‐Lynch suture in posterior PAS may be also explained on the basis that a significant proportion of posterior PAS were discovered only at surgery following extensive bleeding thus inducing the surgeon to undertake these life‐saving measures.

The sub‐analysis comparing cases diagnosed prenatally compared to those detected at the time of surgery showed no significant differences for the main clinical outcome between the two groups, except for the higher rate of hysterectomy in the group with a prenatal diagnosis. These results are difficult to interpret because they are based on a very limited number of cases. Furthermore, in this sub‐analysis, we could not adjust the analysis according to other co‐factors potentially affecting the results, including emergency surgery and severity of PAS.

This is among the largest prospective series exploring the risk factors, accuracy of prenatal diagnosis, and outcome of pregnancies complicated by posterior PAS disorders. The main strengths of the present study are its prospective design, inclusion of cases diagnosed prenatally and receiving a shared management among the different centers, the use of a standardized approach to collect and evaluate ultrasound markers of PAS, and its multicentric nature, which are likely to increase the external validity of our findings. A limitation of this study is its potentially low generalizability, as we evaluated a high‐risk population in the third trimester of pregnancy, with a high overall prevalence of PAS. The small number of cases affected by PAS and confirmed at histopathology did not allow us to identify less common risk factors potentially associated with these anomalies and also affected the computation of the diagnostic accuracy of ultrasound in predicting these disorders. Confirmation of PAS was also affected by the fact that not all patients with posterior PAS had surgery, and histopathological analysis in those not undergoing hysterectomy was based upon assessment of the placental basal plate, thus potentially overestimating the occurrence of PAS in this sub‐set of women. We also could not objectively compare the performance of MRI in detecting posterior PAS in view of the very small number of included cases or that or ultrasound according to gestational age at assessment. Another limitation of the present study is that the imaging protocol adopted when the study was started did not include the number of ultrasound signs recommended by the recent Delphi consensus on prenatal diagnosis of PAS disorders, and this may have affected the diagnostic accuracy of ultrasound in detecting PAS reported in the present cohort. Furthermore, we did not have the long‐term surgical outcome of patients affected by posterior PAS, and we could not compare it with those with anterior PAS. Prenatal MRI was performed only in a very limited number of patients and in a blinded way, thus precluding an objective comparison with ultrasound. We could also not build a reliable predictor model for identifying patients at high probability of posterior PAS in view of the small number of true positive cases and lack of association of most of the maternal, pregnancy, and imaging characteristics and posterior PAS at univariate analysis (Table 1). Finally, a standardized pathological examination of the placenta was not available in 16.7% of the initial cohort.

Extensive research in the field of PAS disorders has led to a higher comprehension of the mechanisms behind its occurrence. Likewise, advances in prenatal imaging techniques have allowed the construction of predictive models able to identify PAS and stratify its severity.^14^ In the present study, we reported a relatively lower diagnostic performance of ultrasound in detecting posterior PAS, while only seven cases underwent MRI, thus making it impossible to extrapolate robust evidence on its role in identifying these anomalies. Identification of posterior PAS implies a careful assessment of the posterior uterine wall and its relationship with placental implantation. Future studies should report specific ultrasound signs for identifying patients at high probability of posterior PAS and should also elucidate whether patients with risk factors for posterior PAS should routinely undergo placental MRI.

## 5 CONCLUSION

In patients with placenta previa, posterior PAS is associated with prior uterine surgery and CS and commonly presents with less severe types of abnormal implantation compared to anterior PAS. Prenatal ultrasound has a low diagnostic accuracy in detecting posterior compared to anterior PAS disorders. Finally, posterior PAS was associated with a lower risk of hysterectomy compared to anterior PAS. Further large multicenter studies are needed to confirm the findings of this study, identify less common risk factors potentially associated with PAS, and develop multiparametric prediction models able to identify these anomalies more accurately.

## AUTHOR CONTRIBUTIONS

Alessandro Lucidi and Francesco D'Antonio designed the study. Francesco D'Antonio and Asma Khalil wrote the paper. All the others provided data.

## CONFLICT OF INTEREST STATEMENT

No conflict of interest to declare by any of the authors.

## ETHICS STATEMENT

The study was registered with ClinicalTrials.gov (NCT02442518). The study was approved by the ethical committee of the coordinating center (University of Brescia; Protocol Number 1837, date of approval November 4, 2014). Ethics approval from each of the collaborating centers was obtained (data available on file and can be provided upon request). Written informed consent was obtained from all study participants.

## Supporting information


Table S1.

Table S2.

## APPENDIX A

The ADoPAD (Antenatal Diagnosis of Placental Attachment Disorders) study group: Alessandro Lucidi^1^, Nicola Fratelli^2^, Claudia Maggi^2^, Cecilia Cavalli^2^, Andrea Sciarrone^3^, Danilo Buca ^1^, Anna Garofalo^3^, Elsa Viora^3^, Patrizia Vergani^4^, Sara Ornaghi^4^, Marta Betti^5^, Isadora Vaglio Tessitore^4^, Anna Franca Cavaliere^6^, Silvia Buongiorno^6^, Annalisa Vidiri^6^, Elisa Fabbri^7^, Enrico Ferrazzi^8,9^, Valeria Maggi^8^, Irene Cetin^7^, Tiziana Frusca^10^, Tullio Ghi^11^, Christine Kaihura^10^, Elvira Di Pasquo^10^, Tamara Stampalija^12,13^, Chiara Belcaro^12^, Mariachiara Quadrifoglio^12^, Michela Veneziano^14^, Federico Mecacci^15^, Serena Simeone^15^, Anna Locatelli^16^, Sara Consonni^17^, Nicola Chianchiano^18^, Francesco Labate^19^, G. Calcagno^19^, Antonella Cromi^20^, Fabio Ghezzi^20^, Emma Bertucci^21^, Fabio Facchinetti^21^, Giulia Andrea Giuliani^21^, Anna Fichera^2^, Daniela Granata^22^, Francesca Foti^23^, Laura Avagliano^24^, Gaetano Pietro Bulfamante^24^, Asma Khalil^25^, Maria Elena Flacco^26^, Lamberto Manzoli^27^, Federico Prefumo^28^, Giuseppe Calì^29^, Francesco D'Antonio^1^

^1^Center for Fetal Care and High‐Risk Pregnancy, Department of Obstetrics and Gynecology, University of Chieti, Chieti, Italy. ^2^Division of Obstetrics and Gynecology, ASST Spedali Civili, Department of Clinical and Experimental Sciences, University of Brescia, Brescia, Italy. ^3^Obstetrics‐Gynecological Ultrasound and Prenatal Diagnosis Unit, Department of Obstetrics and Gynecology, Città della Salute e della Scienza, Turin, Italy. ^4^University of Milan‐Bicocca, School of Medicine and Surgery, Department of Obstetrics and Gynecology, Fondazione MBBM Onlus, San Gerardo Hospital, Monza, Italy. ^5^Obstetrics and Gynaecology Unit, A. Manzoni Hospital, ASST Lecco, Lecco, Italy. ^6^Dipartimento Scienze della Salute della Donna e del Bambino e di Sanità Pubblica, Fondazione Policlinico Universitario ‘A. Gemelli’ IRCCS‐Università Cattolica del Sacro Cuore, Rome, Italy. ^7^Obstetrics and Gynecology Unit, Buzzi Children's Hospital, University of Milan, Milan, Italy. ^8^Fondazione IRCCS Ca Granda Ospedale Maggiore Policlinico, Milano, Unit of Obstetrics, Milan, Italy. ^9^Department of Clinical and Community Sciences, University of Milan, Milan, Italy. ^10^Department of Medicine and Surgery, Obstetrics and Gynaecology Unit, University of Parma, Parma, Italy. ^11^Department of Medicine and Surgery, University of Parma, Parma, Italy. ^12^Unit of Fetal Medicine and Prenatal Diagnosis, Institute for Maternal and Child Health, IRCCS Burlo Garofolo, Trieste, Italy. ^13^Department of Medical, Surgical and Health Science, University of Trieste, Trieste, Italy. ^14^Obstetrics and Gynecology Unit, Bolzano Hospital, Bolzano, Italy. ^15^Department of Woman and Child's Health, Careggi University Hospital, Florence, Italy. ^16^University of Milan‐Bicocca, School of Medicine and Surgery, Obstetrics and Gynecology Unit, Carate Brianza Hospital, ASST Brianza, Carate Brianza, Italy. ^17^Obstetrics and Gynecology Unit, Carate Brianza Hospital, ASST Brianza, Carate Brianza, Italy. ^18^Fetal Medicine Unit, Bucchieri La Ferla‐Fatebenefratelli Hospital, Palermo, Italy. ^19^Department of Obstetrics and Gynaecology, Azienda Ospedaliera Villa Sofia Cervello, Palermo, Italy. ^20^Department of Medicine and Surgery, University of Insubria, Varese, Italy. ^21^Obstetrics and Gynecology Unit, Department of Medical and Surgical Sciences for Children and Adults, University of Modena and Reggio Emilia School of Medicine, Modena, Italy. ^22^Obstetrics and Gynecology Unit, Bolognini Hospital, Seriate, Italy. ^23^Obstetrics and Gynecology Unit, Civico Hospital of Partinico, Palermo, Italy. ^24^Department of Health Sciences, Università degli Studi di Milano, Milan, Italy. ^25^Fetal Medicine Unit, Liverpool Women's Hospital, University of Liverpool, Liverpool, UK. ^26^Department of Environmental and Preventive Sciences, University of Ferrara, Italy. ^27^Department of Medical and Surgical Sciences, University of Bologna, Italy. ^28^Obstetrics and Gynecology Unit, IRCCS Istituto Giannina Gaslini, 16147 Genova, Italy. ^29^Department of Obstetrics and Gynaecology, Arnas Civico Hospital, Palermo, Italy.
