# Supplementary material for: Risk factors, prenatal diagnosis, and outcome of posterior placenta accreta spectrum disorders in patients with placenta previa or low‐lying placenta: A multicenter study
Source: Acta Obstet Gynecol Scand. 2025 May 19;104(7):1328–38. doi: 10.1111/aogs.15132 (PMC12144582; doi:10.1111/aogs.15132)
Supplement: Supplementary file 1 — Table S1. [file AOGS-104-1328-s002.docx]

**Table S1**. Selected demographic and clinical characteristic to predict a diagnosis of posterior placenta accreta spectrum (PAS) disorder: summary estimates of sensitivity, specificity, positive and negative predictive values (PPV and NPV), positive and negative likelihood ratios (LR+ and LR-) and diagnostic odds ratios (DOR).

| ***Clinical characteristics*** | **Sensitivity**  **% (95% CI)** | **Specificity**  **% (95% CI)** | **PPV**  **% (95% CI)** | **NPV**  **% (95% CI)** | **LR+**  **(95% CI)** | **LR-**  **(95% CI)** | **DOR**  **(95% CI)** |
| --- | --- | --- | --- | --- | --- | --- | --- |
|  |  | | | | | | |
| BMI>30 | 14.3  (1.78-42.8) | 92.2  (88.1-95.2) | 9.52  (1.17-30.4) | 94.9  (91.3-97.4) | 1.83  (0.47-7.10) | 0.93  (0.73-1.15) | 1.97  (0.00-8.56) |
|  |  |  |  |  |  |  |  |
| Multigravidity | 8.99  (5.23-14.2) | 93.8  (86.0-97.9) | 76.2  (52.8-91.8) | 31.6  (25.8-38.0) | 1.44  (0.55-3.79) | 0.97  (0.90-1.04) | 1.48  (0.54-4.04) |
|  |  |  |  |  |  |  |  |
| Multiparity | 9.62  (3.20-21.0) | 92.2  (87.7-95.5) | 23.8  (8.22-47.2) | 80.2  (74.5-85.0) | 1.24  (0.48-3.22) | 0.98  (0.89-1.08) | 1.26  (0.46-3.51) |
|  |  |  |  |  |  |  |  |
| Caucasian ethnicity | 8.02  (4.74-12.5) | 91.3  (79.2-97.6) | 81.0  (58.1-94.6) | 17.7  (13.1-23.2) | 0.93  (0.33-2.61) | 1.01  (0.91-1.11) | 0.92  (0.31-2.72) |
|  |  |  |  |  |  |  |  |
| Prior CS | 20.6  (11.5-32.7) | 95.9  (92.1-98.2) | 61.9  (38.4-81.9) | 78.9  (73.2-83.9) | 5.03  (2.19-11.6) | 0.83  (0.73-0.94) | 6.08  (2.44-15.1) |
|  |  |  |  |  |  |  |  |
| Prior curettage | 6.74  (2.51-14.1) | 91.1  (85.8-94.9) | 28.6  (11.3-52.2) | 65.0  (58.5-71.0) | 0.76  (0.31-1.89) | 1.02  (0.95-1.10) | 0.74  (0.29-1.93) |
|  |  |  |  |  |  |  |  |
| Prior myomectomy | 8.70  (1.07-28.0) | 91.9  (87.7-95.1) | 9.52  (1.17-30.4) | 91.1  (86.8-94.4) | 1.08  (0.27-4.33) | 0.99  (0.87-1.13) | 1.08  (0.00-4.50) |
|  |  |  |  |  |  |  |  |
| Placenta previa major | 24.3  (18.4-31.1) | 75.4  (63.5-84.9) | 73.0  (60.3-83.4) | 26.7  (20.6-33.5) | 0.99  (0.61-1.60) | 1.00  (0.85-1.17) | 0.98  (0.52-1.85) |
|  |  |  |  |  |  |  |  |

BMI, body mass index; CS, cesarean section; CI, confidence interval.
