# Supplementary material for: Risk factors, prenatal diagnosis, and outcome of posterior placenta accreta spectrum disorders in patients with placenta previa or low‐lying placenta: A multicenter study
Source: Acta Obstet Gynecol Scand. 2025 May 19;104(7):1328–38. doi: 10.1111/aogs.15132 (PMC12144582; doi:10.1111/aogs.15132)
Supplement: Supplementary file 2 — Table S2. [file AOGS-104-1328-s001.docx]

**Table S2**. Selected demographic and clinical characteristics of the sample, overall and among the women with posterior versus anterior placenta accreta spectrum (PAS) disorders.

|  | **Overall**  **Sample** | **Posterior**  **PAS** | **Anterior**  **PAS** | **p *** |
| --- | --- | --- | --- | --- |
| *Variables* | (N=99) | (N=21) | (N=78) |  |
|  |  |  |  |  |
| *Demographic and clinical characteristics:* |  |  |  |  |
| Mean maternal age at delivery in years (SD) | 35.0 (5.0) | 34.7 (6.1) | 35.0 (4.7) | 0.8 |
|  |  |  |  |  |
| Mean BMI, (SD) | 23.8 (5.3) | 24.4 (6.6) | 23.6 (4.9) | 0.6 |
|  |  |  |  |  |
| Multigravid women, % | 86.9 | 76.2 | 89.7 | 0.10 |
|  |  |  |  |  |
| *Parity status, %* |  |  |  | 0.14 |
| - Nulliparous women | 20.2 | 33.3 | 16.7 |  |
| - Primiparous women | 40.4 | 42.9 | 39.7 |  |
| - Multiparous women | 39.4 | 23.8 | 43.6 |  |
|  |  |  |  |  |
| ART, % | 6.1 | 9.5 | 5.1 | 0.5 |
|  |  |  |  |  |
| Caucasian ethnicity, % | 86.9 | 81.0 | 88.5 | 0.4 |
|  |  |  |  |  |
| Prior CS, % | 77.8 | 61.9 | 82.1 | 0.049 |
|  | (N=41) | (N=7) | (N=34) |  |
| Prior CS during labour, % | 29.3 | 28.6 | 29.4 | 0.6 |
|  |  |  |  |  |
| Prior curettage, % | 37.4 | 28.6 | 39.7 | 0.3 |
|  |  |  |  |  |
| Prior myomectomy, % | 22.2 | 61.9 | 11.5 | <0.001 |
|  |  |  |  |  |
| Single pregnancy, % | 96.9 | 95.0 | 97.4 | 0.6 |
|  |  |  |  |  |
| Major placenta previa, % | 89.9 | 95.7 | 91.0 | 0.5 |
|  |  |  |  |  |
| Posterior PAS disorders, % |  |  |  |  |
| - Accreta | 36.4 | 66.7 | 28.2 | 0.001 |
| - Increta | 19.2 | 23.8 | 17.9 | 0.5 |
| - Percreta | 44.4 | 9.5 | 53.9 | <0.001 |
|  |  |  |  |  |
| Placenta overlapping the internal cervical os, % | 89.1 | 85.0 | 90.3 | 0.5 |
|  |  |  |  |  |
| Mean overlap length in mm (SD) | 32.3 (25.0) | 28.0 (25.9) | 33.5 (24.8) | 0.4 |
|  |  |  |  |  |
| Mean cervical length in mm (SD) | 34.6 (10.1) | 36.1 (8.7) | 34.3 (10.4) | 0.5 |
|  |  |  |  |  |
| Mean placental thickness in mm (SD) | 37.2 (14.3) | 35.1 (17.5) | 37.7 (13.3) | 0.5 |
|  |  |  |  |  |
| *US signs:* |  |  |  |  |
| Interrupted hypoechoic retroplacental space, % | 83.8 | 61.9 | 89.7 | 0.002 |
|  |  |  |  |  |
| Interrupted hyperecogenic bladder line, % | 56.6 | 14.3 | 68.0 | <0.001 |
|  |  |  |  |  |
| Lacunae, % | 68.7 | 61.9 | 70.5 | 0.5 |
|  | (N=25) | (N=8) | (N=17) |  |
| One sign only (vs none), % | 40.0 | 0.0 | 58.8 | 0.005 |
|  | (N=50) | (N=18) | (N=32) |  |
| Two signs (vs ≤1), % | 50.0 | 55.6 | 46.9 | 0.6 |
|  | (N=99) | (N=21) | (N=78) |  |
| Three signs (vs ≤2), % | 49.5 | 14.3 | 59.0 | <0.001 |
|  |  |  |  |  |
| Prenatal US-suspected posterior PAS, % | 91.9 | 61.9 | 100 | <0.001 |
|  |  |  |  |  |
| MRI performed, % (n) | 58.6 (58) | 33.3 (7) | 65.4 (51) | 0.008 |
|  |  |  |  |  |
| MRI-suspected posterior PAS, % | 96.6 | 100 | 96.1 | 0.6 |
|  |  |  |  |  |

* Chi-squared test for categorical variables; T-test and Kruskal-Wallis test for normally distributed and non-normally distributed continuous variables, respectively.

SD, standard deviation; ART, assisted reproductive technique; CS, cesarean section.
